# Endoscopic detachable snare ligation for rebleeding after over-the-scope clip placement in colonic diverticular bleeding: salvage treatment

**DOI:** 10.1055/a-2599-9835

**Published:** 2025-06-03

**Authors:** Takaaki Kishino, Takayuki Sawa, Yoko Kitamura

**Affiliations:** 1 53356 Department of Gastroenterology and Hepatology, Center for Digestive and Liver Diseases, Nara City Hospital, Nara, Japan

The over-the-scope (OTS) clip is a nitinol-based, shape-memory clip used for suturing and hemostasis in the gastrointestinal tract 1 . When used for hemostasis of colonic diverticular bleeding (CDB), the OTS clip preserves blood flow in the grasped tissue, potentially reducing the risk of delayed perforation compared to methods such as endoscopic band ligation 2 . It has shown favorable outcomes as a first-line treatment for CDB 2 . However, data on salvage treatment for rebleeding after OTS clip placement remain limited, with repeated OTS clip application, arterial embolization, and surgery being the primary options 2 3 . This report describes a case in which endoscopic detachable snare ligation was effective for rebleeding after OTS clip placement in CDB.

An 88-year-old woman presented to the emergency department with hematochezia and loss of consciousness. An urgent colonoscopy identified a diverticulum with an exposed vessel in the ascending colon, for which an OTS clip was applied ( Fig. 1 ). However, the hematochezia and vital signs indicating shock recurred on the following day. Repeat colonoscopy confirmed active bleeding from the OTS clip-treated site, and hemostasis was achieved with endoscopic clipping ( Fig. 2 , Video 1 ). Four days post-clipping, there was another episode of hematochezia with vital signs indicating shock. Colonoscopy revealed clip detachment and a clot on the apex of the diverticulum grasped by the OTS clip ( Fig. 3 ). Placing a detachable snare over the OTS clip was challenging; however, by inserting the tip of the snare into a gap within the OTS clip, the snare was stabilized, allowing for successful ligation of the diverticulum ( Fig. 3 , Video 1 ). The patient was discharged 4 days later without further rebleeding.

**Fig. 1:**
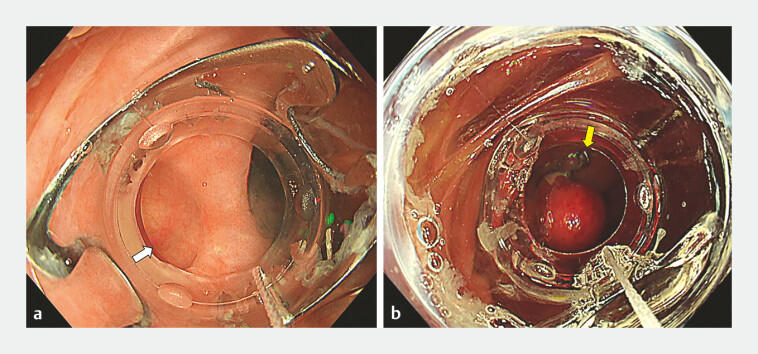
**a** A diverticulum with an exposed vessel (white arrow) in the ascending colon. **b** An OTS clip (yellow arrow) deployed to the affected diverticulum, inverting and fixing it. Abbreviation: OTS, over-the-scope.

**Fig. 2:**
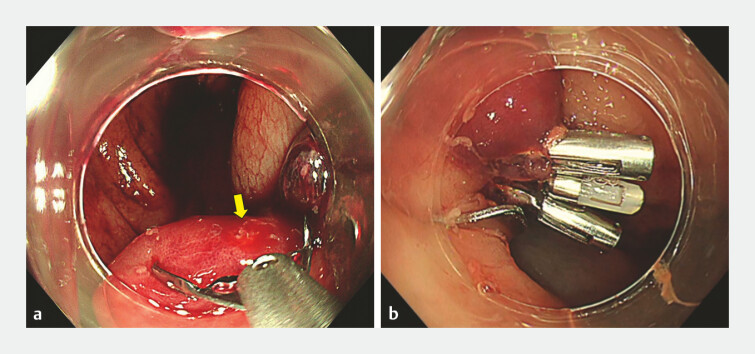
**a** Colonoscopy performed after early rebleeding following primary hemostasis with an OTS clip. The OTS clip remains in place, maintaining the diverticulum in an inverted and bulging state, but active bleeding (yellow arrow) is observed from the apex. **b** Clipping was performed to stop the active bleeding, achieving temporary hemostasis. Abbreviation: OTS, over-the-scope.

**Fig. 3:**
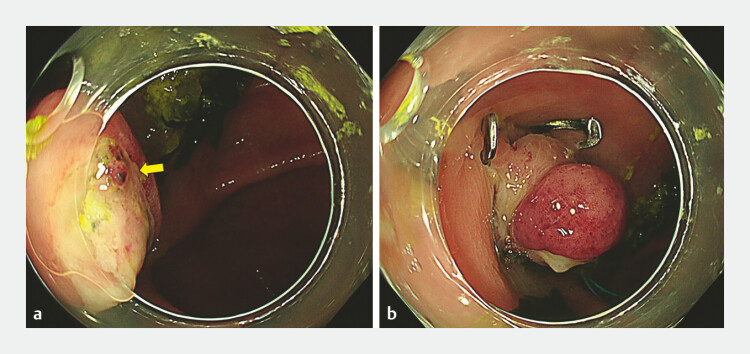
Colonoscopy for rebleeding after clipping. **a** A clot is observed at the apex of the OTS clip-inverted diverticulum (yellow arrow). **b** The OTS clip-inverted diverticulum is ligated using a detachable snare. Abbreviation: OTS, over-the-scope.

Detachable snare ligation for an OTS clip-inverted colonic diverticulum.Video 1

Rebleeding after OTS clip placement poses a therapeutic challenge. Endoscopic detachable snare ligation may be a feasible salvage option in such cases and potentially in other gastrointestinal bleeding sites following OTS clip use.

Endoscopy_UCTN_Code_TTT_1AQ_2AZ

## Notes

Endoscopy E-Videos https://eref.thieme.de/e-videos
*E-Videos* is an open access online section of the journal *Endoscopy* , reporting on interesting cases and new techniques in gastroenterological endoscopy. All papers include a high-quality video and are published with a Creative Commons CC-BY license. Endoscopy E-Videos qualify for HINARI discounts and waivers and eligibility is automatically checked during the submission process. We grant 100% waivers to articles whose corresponding authors are based in Group A countries and 50% waivers to those who are based in Group B countries as classified by Research4Life (see: https://www.research4life.org/access/eligibility/ ). This section has its own submission website at https://mc.manuscriptcentral.com/e-videos .
